# Examining the Link Between Physical Activity and Cognitive Function: A Parallel Mediation Model of Health and Wellbeing Among Adolescents

**DOI:** 10.3389/fpsyg.2022.764842

**Published:** 2022-03-02

**Authors:** Xi Luan, Ji Liu, Xin Luo

**Affiliations:** Faculty of Education, Shaanxi Normal University, Xi’an, China

**Keywords:** moderate physical activity, subjective wellbeing, general health, PISA, structural equation modeling

## Abstract

**Background:**

Adolescents’ engagement in daily physical activity brings multiple benefits, including reduction in obesity, improvement of mental health, and enhancement of cognitive function (CF). While prior studies have examined the link between physical activity and cognitive function, little is known regarding the extent to which this relationship is shaped by health and wellbeing factors. This study examines how subjective wellbeing (SWB) and general health (GH) mediate the relationship between adolescents’ physical activity and cognitive function.

**Methods:**

This study estimates a parallel structural equation model using the Program for International Student Assessment 2018 dataset. Specifically, a total of 63,228 15-year-old subjects in nine countries/economies satisfied the study inclusion criteria, including in Bulgaria, Georgia, Hong Kong, Ireland, Mexico, Panama, Serbia, Spain, and United Arab Emirates. Frequency of moderate physical activity (MPA, ≥3.0 Metabolic Equivalent Task) was reported weekly; SWB and GH were assessed using an internationally validated multi-item standardized questionnaire. SWB was measured by students’ self-evaluated satisfaction with their health, life, and schooling. GH was measured by students’ physical health and mental health status. Cognitive function (CF) was modeled as a latent function consisting of plausible values derived using item response theory on reading, mathematics, and science achievement tests.

**Results:**

Findings indicated that increase in weekly MPA was positively associated with higher levels of SWB (*p* < .001), GH (*p* < .001), and CF (*p* < .001) among the study subjects. Parallel mediation analyses revealed that more frequent weekly MPA had relatively large direct effects (*p* < .001) on CF, and indirect effects channeling through improvements in SWB and GH were non-trivial (*p* < .001). Heterogeneity results showed that boosts to CF, associated with MPA, were larger for mathematics and science than for reading (*p* < .001).

**Conclusion:**

This study used a large-scale international dataset to show that the positive relationship observed between MPA and CF among adolescents was robust, and that SWB and GH were two critical mediators through which physical activity positively bolster CF.

## Introduction

Physical activity is defined as bodily movement that involves skeletal muscles and energy expenditure (Physical Activity Guidelines Advisory Committee, 2018; Sneck et al., 2019). Adolescents are advised by the World Health Organization (WHO) to participate in at least 60 min of moderate physical activity (MPA) each day in order to stay healthy (World Health Organization, 2010). This volume of physical activity is also recommended by U.S. Department of Health and Human Services (2012), citing its instrumental influence on obesity prevention. However, as academic pressure encroaches on adolescents’ life, engagement in MPA has rapidly declined while increase in sedentary behavior has been widely observed (Van Stralen et al., 2014; Hartikainen et al., 2021). This trend, evidenced in recent literature, is likely to pose detrimental risks to adolescents’ health, wellbeing, and cognitive function (Corder et al., 2015; Hale et al., 2021).

With regard to adolescents’ health and wellbeing, the decreasing level of engagement with MPA negatively influences their musculoskeletal development, diet, sleep, body mass index (BMI), and sensory-motor interaction (Have et al., 2016; Zayed and Jansen, 2018; Barbosa et al., 2020). In addition, studies show that the reduction of MPA involvement can have adverse impacts on adolescents’ life satisfaction, self-image, and self-acceptation (Chen et al., 2021). Worryingly, there is a global trend of adolescents’ increasing sedentary behavior, which leads to the decline in their MPA involvement (Hale et al., 2021). Accordingly, this is likely to hinder their cognitive, motor, and social development, and is related to lower level of cognitive function (CF) among adolescents due to narrowing chances for neurogenesis, angiogenesis, and enhancement of central nervous system metabolism (Barbosa et al., 2020). As a result, adolescents’ executive function may be impacted (Singh et al., 2019). To address these concerns, previous studies have investigated the relationship between MPA involvement and CF, and have found that adolescents’ general health (GH) and subjective wellbeing (SWB) benefit from MPA and are both important factors in determining CF (Aadland et al., 2017; Barbosa et al., 2020).

In terms of mechanisms of how MPA influences adolescents’ CF, existing evidence has identified three lines of potential channels, including physiological factors, executive function, and learning dispositions. As for the physiological factors, routine MPA is associated with the increase of brain-derived neurotrophic factor, insulin-like growth factor-1 and vascular endothelial growth factor, all of which are strong determinants of developmental growth, maintenance and plasticity of the brain, and are related to CF (Barbosa et al., 2020). Neural growth and enhancement of synaptic transmission can lead to changes in adolescents’ prefrontal cortex and improvement of their executive functions including abstract reasoning, planning, and problem solving (Kopp, 2012). In addition, different types of MPA are found to influence CF in different ways. Some MPA can increase physiological arousal, leading to better attention and the release of neurotransmitters, whereas more aerobic MPA improves cardiovascular fitness and ameliorate oxygen saturation and glucose delivery, bringing benefits to neurogenesis and angiogenesis in brain areas that are responsible for CF (Egger et al., 2019; Singh et al., 2019). Besides influencing core executive function, benefits of MPA on adolescents’ CF are also mediated by the cultivation of learning dispositions and learning behaviors during their engagement of MPA which can help them excel in CF tests. The learning dispositions and qualities include organization skills, on-task behaviors and improved school attendance (Álvarez-Bueno et al., 2016).

More importantly, it has been well documented that adolescents’ MPA benefits both their physical and mental health (Penedo and Dahn, 2005; Barbosa et al., 2020), thus enhancing the level of their general health (GH) (Carson et al., 2016). On the one hand, in regard to physical health, MPA can ameliorate adolescents’ visual-motor coordination and improve their cardiovascular fitness (Cosgrove et al., 2018). MPA can also keep adolescents’ physically fit by reducing the chance of obesity and cardio-metabolic disease (Zayed and Jansen, 2018). MPA is also associated with a healthy diet, better sleep and can enhance adolescents CF (Cosgrove et al., 2018). On the other hand, as for the mental health, MPA is related with positive self-image, self-esteem, and self-efficacy. It can effectively reduce depressive symptomatology, psychological stress, and anxiety (Carson et al., 2016; Hale et al., 2021). Adolescents’ physical and mental health can in turn benefit cognitive performance and brain development, laying the physiological foundations for CF (Haverkamp et al., 2020). The lack of MPA is, therefore, seen as a major threat for adolescents’ GH and CF.

Additionally, subjective wellbeing (SWB) is a critical dimension of adolescents’ wellbeing and is a key indicator reflecting quality of life. It is a notion that describes people’s life satisfaction, pleasant and unpleasant affect (Hale et al., 2021). Research shows that SWB has close relationship with people’s sports involvement. Body image, self-esteem, and personal experience in sports can pose as barriers in sports participation (Martin et al., 2015). Likewise, regular exercise can improve life satisfaction, reduce depressive symptoms and improve mood (Jodra and Domínguez, 2020). By helping people gain social integration, social support and a sense of belonging, MPA is associated with self-acceptation, positive relationship, personal growth, environmental mastery, and purpose of life (Chen et al., 2021).

Despite the above discussions on adolescents’ MPA, GH, SWB and CF, there are important gaps needed to fill. Previous research mainly focused on acute physical activity interventions in pre-adolescent children (Haverkamp et al., 2020) and there is a relative lack of research on adolescents and on the effects of regular physical activity (Verburgh et al., 2013; Li et al., 2017; Xue et al., 2019). Furthermore, while most studies examined the effects of MPA on CF, few studies described its effects on CF embodied by the specific fields of language, math, and science (Haapala, 2012). Also, as previous research mainly used interventional approach or conducted metanalysis of the existing study (Egger et al., 2019; Haverkamp et al., 2020), large scale and cross-cultural studies are needed. Finally, little is known about whether GH and SWB, two significant factors benefited from MPA, can mediate the effects of MPA on adolescents’ CF.

## Materials and Methods

### Constructs and Measures

This study leveraged the publicly-available Program for International Student Assessment (PISA) 2018 dataset,^1^ which is the flagship project of the Organization for Economic Cooperation and Development (OECD) and sampled 612,004 15-year-old students in more than 70 economies (Liu and Steiner-Khamsi, 2020). PISA used a multi-stage and multi-strata sampling design, for which schools were sampled with students aged 15–16 at the time of assessment (OECD, 2020). In the first stage, schools were selected using a probability-proportional-to-size sampling approach from a systematic list of eligible schools within participating economies. In the second stage, students were randomly selected from a complete list of eligible 15-year-old students within each sampled school. In terms of data collection, each sampled student provided detailed background information and completed CF assessment on math, reading, and science competency, in addition to filling a separate youth wellbeing assessment questionnaire which encompassed a series of questions covering life satisfaction, health status, and physical activity.

Program for International Student Assessment contains tests on key cognitive domains and is widely used as an instrument to assess and compare students’ school performance and learning dispositions across different countries and regions (Lee and Stankov, 2018). The major cognitive domains in PISA are reading, math and science (Liu, 2019; OECD, 2020), as the knowledge and skills embodied in these domains are widely seen as indicators for the successful participation in contemporary societies and are key to the sustainable growth of modern economy (Heckman and Jacobs, 2009; OECD, 2019; Araújo et al., 2020). Reading domain in PISA tests an individual’s capacity to understand, use, evaluate, reflect on and engage with texts to achieve one’s goals, develop one’s knowledge and potential, and participate in society; math is to examine students’ competence to analyze, reason and communicate ideas effectively as they pose, formulate, solve and interpret solutions to mathematical problems; and science domain deals with students’ ability to engage with science-related issues, to reason with the ideas of science, as a reflective citizen (OECD, 2020). The results of the cognitive domains in PISA tests attract wide public attention as they offer insights, from a comparative perspective, into the effectiveness of educational policy making and provide large-scale, cross-cultural data for research analysis (Araújo et al., 2017).

Apart from the cognitive domains, two key constructs in this study, SWB and GH, are anchored on the PISA 2018 wellbeing survey. In broad strokes, the PISA wellbeing questionnaire was designed to maximize cross-cultural comparability by choosing clear, translatable, quantifiable, and vignette-based Likert-scale items (OECD, 2019). This study leveraged data availability of students’ frequency of engagement in MPA, level of SWB and GH, as well as CF assessment in math, reading, and science. Below, a more detailed descriptions of the predictor variable, mediator variables, and outcome variables are to be reported.

Firstly, MPA [≥3.0 Metabolic Equivalent Task (MET)] was the predictor variable in this study. In PISA, MPA is referred as physical activities including but not limited to walking, climbing stairs, riding bicycles, for a total of at least 60 min per day. Respondents in PISA reported how many days they engaged in these physical activities during the past 7 days.

Secondly, SWB and GH, the two mediator variables in this study, were assessed using an internationally validated multi-item standardized Likert-scale questionnaire in PISA. For SWB, respondents answered ten Likert-scale questions regarding their self-evaluated satisfaction with health, life, and school. The 10 questions are “Your health; the way that you look; what you learn at school; the friends you have; the neighborhood you live in; all the things you have; how you use your time; your relationship with your parents/guardians; your relationship with your teachers; your life at school.” For GH, respondents answered nine Likert-scale questions on how often they had some physical or mental health status, including headache, stomach pain, back pain, feeling depressed, irritability or bad temper, feeling nervous, difficulties in getting to sleep, feeling dizzy, and feeling anxious.

Thirdly, CF as the outcome variable was modeled as a latent construct. In PISA’s cognitive assessment, emphasis was put on respondents’ complex CF, particularly on individuals’ capacity to understand, evaluate, formulate, reason, and reflect (OECD, 2018). It was measured by performance in mathematics, reading, and science domains. Leveraging item response theory, ten plausible values were generated for each respondent per subject, and a probability distribution for a student’s ability is estimated instead of a direct estimation of a student’s ability (OECD, 2009).

### Hypotheses

**Figure 1:**
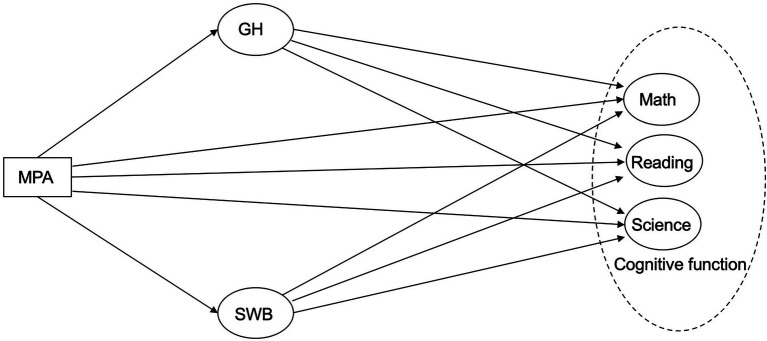
Conceptual diagram.

Based on the discussions of the relationships between MPA, GH, SWB, and CF, the following hypothesis were made (see Figure 1): adolescents’ MPA influences CF (embodied by their performance in PISA math, reading, and science tests), and that GH and SWB, mediate the effects of MPA on adolescents’ CF.

### Sample Characteristics

The inclusion criteria for study subjects were as follows: (1) reported frequency of MPA (≥3.0 MET); (2) reported SWB and GH using multi-item standardized Likert-scale; and (3) completed cognitive test results for math, reading, and science. A total of 63,228 subjects satisfied the inclusion criteria, and the descriptive statistics for the participating students were presented in Table 1. These 63,228 students are from nine countries and economies, including Bulgaria, Georgia, Hong Kong, Ireland, Mexico, Panama, Serbia, Spain, and United Arab Emirates. Among these students, 32,562 (51.5%) were female. The participants’ age ranged from 15.812 to 15.816 (*M* = 21.26, SD = .29). On average, these students in the sample reported 3.168 times of MPA. The average scores of their test results on math, reading, and science were 474.004, 471.488, and 470.640, respectively.

**Table 1:** Demographic information of participants and descriptions of predictor and outcome variables (*N* = 63,228).

Variable	Mean	SD	95% CI	
			Lower	Upper
Female	.515	–	–	–
Age	15.814	.290	15.812	15.816
MPA	3.168	.010	3.149	3.186
Math (10 plausible values)	474.004	98.867	375.137	572.871
Reading (10 plausible values)	471.488	101.444	370.044	572.932
Science (10 plausible values)	470.640	97.026	373.614	567.666

### Statistical Analysis

The study utilized structural equation modeling (SEM) to examine the relationship between MPA and CF, and fitted a parallel mediation model to evaluate the extent to which SWB and GH mediated their relationship. In recent decades, SEM has grown in popularity in scientific inquiry, especially among new applications in educational psychology research (Karimi and Meyer, 2014). Methodologically speaking, SEM allows the researcher to statistically examine the extent to which proposed hypotheses are supported by empirical data to reflect theoretical predictions (Kline, 2015).

This present study utilized partial least squares SEM model (PLS-SEM). All analyses were performed using STATA version 15.0 (Stata, StataCorp LLC, College Station, TX, United States) software. Several goodness of fit indices was tested to evaluate how well the structural models fitted the data. The comparative fit index was computed to be .912, while Tucker-Lewis index was computed to be .905. Both were higher than .9, suggesting that the data displayed a reasonable fit for the model (Bentler, 1990). The value of Root Mean Square Error of Approximation was .104, which was marginally above the goodness-of-fit threshold of .10 (McDonald and Marsh, 1990).

## Results

### Measurement Model

While PISA 2018 has already categorized survey items by broad construct groupings, we report psychometric properties of presumed scales and assess measurement model fit (Matsunga, 2010). Tables 2 and 3 presented results from the measurement models, with information on factor loadings. Importantly, all item loading values were above .60, showing good explanation of each factor, which indicated good internal consistency and construct validity (Hair et al., 2010, 2016). The internal consistency of both variables was assessed using Cronbach’s *α*, which was .892 for SWB and .880 for GH. In Table 4, we reported correlation coefficients between constructs, and found that all construct pairs exhibited significant and positive associations.

**Table 2:** Reliability and convergent validity of subjective wellbeing (*N* = 63,228).

Items	Factor loadings
1. Your health	.691
2. The way that you look	.694
3. What you learn at school	.691
4. The friends you have	.699
5. The neighborhood you live in	.699
6. All the things you have	.756
7. How you use your time	.711
8. Your relationship with your parents/guardians	.735
9. Your relationship with your teachers	.721
10. Your life at school	.751

**Table 3:** Reliability and construction validity of general health (*N* = 63,228).

Items	Factor loadings
1. Headache	.688
2. Stomach pain	.673
3. Back pain	.627
4. Feeling depressed	.757
5. Irritability or bad temper	.753
6. Feeling nervous	.749
7. Difficulties in getting to sleep	.699
8. Feeling dizzy	.745
9. Feeling anxious	.754

**Table 4:** Correlation matrix of SEM input measures (*N* = 63,228).

	1	2	3	4	5	6
1. MPA	–					
2. SWB	.072^***^	–				
3. GH	.051^***^	.351^***^	–			
4. Math	.093^***^	.114^***^	.152^***^	–		
5. Reading	.071^***^	.116^***^	.097^***^	.829^***^	–	
6. Science	.088^***^	.114^***^	.135^***^	.845^***^	.881^***^	–

*** *p* < .001.

### Structural Equation Modeling

The estimation of standardized direct effects was reported in Table 5. MPA had significant positive effects on both GH and SWB. The path coefficient from MPA to GH was significant (.189, *p* < .001), and so was the path coefficient from MPA to SWB (.183, *p* < .001). That is, both GH and SWB were influenced by students’ MPA.

**Table 5:** Standardized direct effects of the SEM model (*N* = 63,228).

Pathways	Std. coefficient	SE	Value of *p*	95% CI	
				Lower	Upper
MPA→GH	.189	.014	.000	.163	.216
MPA→SWB	.183	.009	.000	.165	.202
MPA→Math	.036	.001	.000	.034	.039
MPA→Reading	.027	.001	.000	.024	.030
MPA→Science	.034	.001	.000	.031	.037
GH→Math	.013	.000	.000	.012	.014
GH→Reading	.006	.000	.000	.005	.007
GH→Science	.011	.000	.000	.010	.012
SWB→Math	.004	.001	.000	.003	.005
SWB→Reading	.008	.001	.000	.007	.009
SWB→Science	.005	.001	.000	.004	.007

CFI = .912; TLI = .905; RMSEA = .104; *χ*^2^(487) = 37,8631.66.

CF was assessed using three cognitive test domains in PISA. MPA was positively related to students’ scores on math. The path coefficient from MPA to math was .036 (*p* < .001). This result demonstrated that each additional MPA per week was associated with .036 more standardized scores on math. Students’ performance on reading was also positively influenced by their MPA (.027, *p* < .001). Moreover, positive effect was in correlation with MPA on the subject of science. The path coefficient from MPA to science was .034 (*p* < .001), indicating that each additional MPA active day was related to .034 higher standard deviations on CF in math.

GH showed direct effects on CF. According to the model, the path coefficient from GH to math was significant (.013, *p* < .001). Meanwhile, the path coefficient from GH to reading was also positive and significant (.006, *p* < .000). It can be also observed that GH had positive and significant effects on students’ achievements on science tests in PISA (.011, *p* < .001). The results suggested that students who reported higher level of GH were likely to achieve more on math, reading, and science.

Subjective wellbeing had a positive and significant effect on math (.004, *p* < .001). From SWB to reading, there was also a direct path with a significant path coefficient (.008, *p* < .001). Moreover, it can be also observed that SWB (.005, *p* < .001) had a positive and significant effect on students’ science tests in PISA. Therefore, students’ higher level of SWB was associated with better CF.

According to the model, a consistent pattern was observed among math, reading, and science. Students’ CF on math, reading, and science was all found to be related to MPA, while there were differences on the path coefficients. The results of the model suggested that each additional MPA per week indicated additional .036 standard scores on math, .034 standardized scores on science, and .027 standardized scores on reading. Furthermore, GH was demonstrated to have positive and significant effects on all the subjects. Similarly, the positive effect of GH was comparatively higher on math (.013) and science (.011), and lower on reading (.006). In the meantime, all the three subjects were directly associated with the variable of SWB. Whereas, students with higher level of SWB tended to score higher on reading (.008, *p* = .000), math (.004, *p* = .000), and science (.005, *p* = .000).

**Figure 2:**
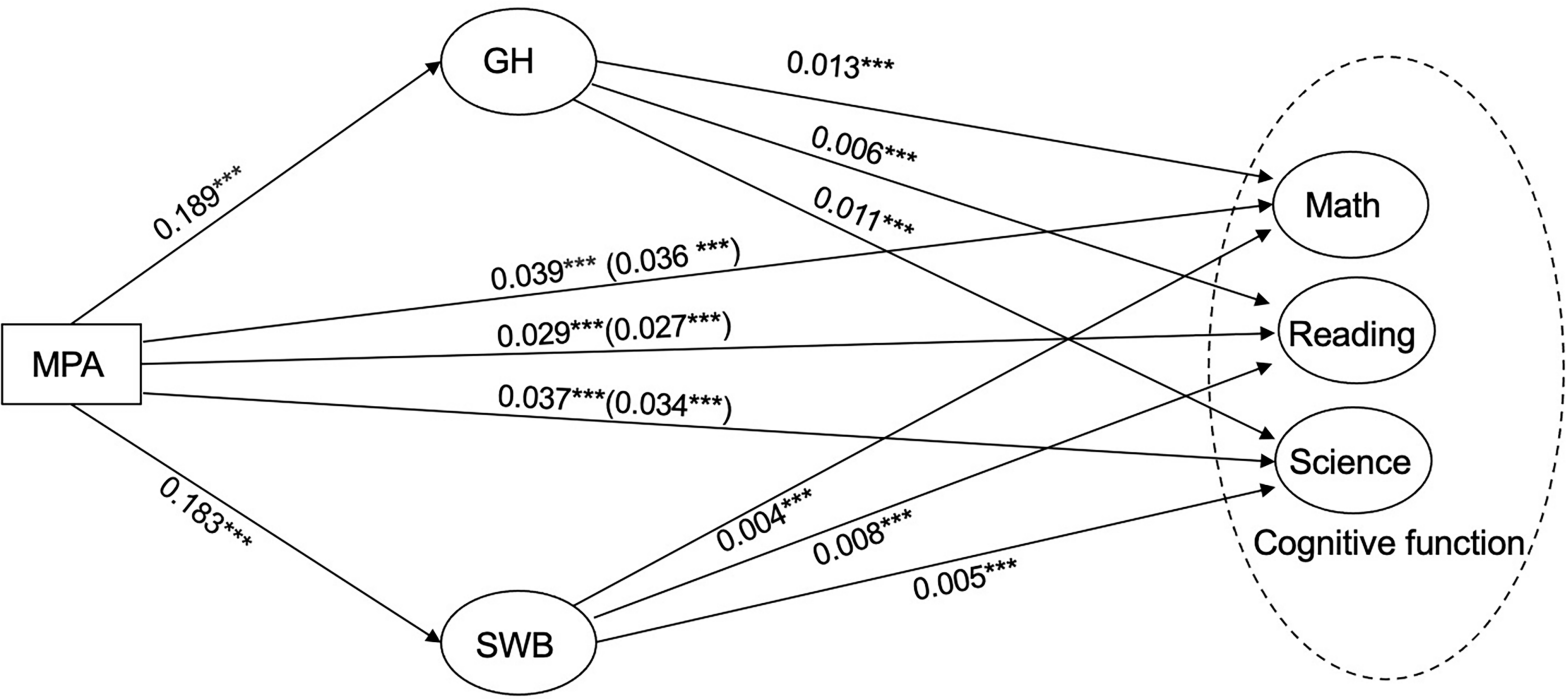
The full parallel mediation model. ***p<0.001, parenthetical=after adding mediating variable coefficients.

The analysis above addressed the direct effect of MPA on CF, the influence of the mediators, GH and SWB, is shown in Figure 2 and Table 6 and is to be reported below. Firstly, as for the effects of MPA on math, this study tested the indirect effects *via* the influence of SWB and GH respectively. Both SWB (standardized indirect effect = .001) and GH (standardized indirect effect = .003) were simultaneously. Both indirect path coefficients tested in our models were positive and significant. Combined with the direct effect (direct effect = .036), the total effect of MPA on math was .039.

**Table 6:** Standardized indirect effects and 95% confidence intervals for the model (*N* = 63,228).

Pathways	Std. coefficient	SE	Value of *p*	95% CI	
				Lower	Upper
MPA→GH→Math	.003	.000	.000	.002	.003
MPA→SWB→Math	.001	.000	.000	.001	.001
MPA→Math	.003	.000	.000	.003	.004
MPA→GH→Reading	.001	.000	.000	.001	.001
MPA→SWB→Reading	.002	.000	.000	.001	.002
MPA→Reading	.003	.000	.000	.002	.003
MPA→GH→Science	.002	.000	.000	.002	.002
MPA→SWB→Science	.001	.000	.000	.001	.001
MPA→Science	.003	.000	.000	.003	.004

Secondly, concerning the effects of MPA on reading, MPA had indirect effects on reading through GH (standardized indirect effect = .001) and through SWB (standardized indirect effect = .002) respectively. Both these indirect path coefficients in models were positive and significant. Thus, with the direct effect (.027), the total effect of MPA on reading was computed as .029.

Lastly, as for the effects of MPA on science, both GH and SWB were found to be mediators (standardized indirect effects are .002 and .001 respectively). The total indirect effect was .003. Adding to the direct effect of MPA on science (direct effect = .034), the total effects of MPA on science were .037.

## Discussions and Conclusions

This study investigated the effects of MPA on student’s CF. Existing research have demonstrated the significance of MPA on CF. Yet there is a lack of study focusing on the effects of MPA among adolescents and the mediating roles of GH and SWB. To fill the gap, this study explored the direct effects of MPA on adolescents’ CF based on large-scale, cross-cultural data and examined the mediating channels of GH and SWB.

Our findings demonstrated that MPA had both direct and indirect positive effects on students’ CF. The path coefficient from MPA to GH was .189 (*p* < .001), while the path coefficient from MPA to SWB was .183 (*p* < .001). MPA was found to have positive direct effects on math (path coefficient = .036, *p* < .001), reading (.027, *p* < .001), and science (.034, *p* < .001). GH and SWB were found to play significant mediating roles. Through the mediation of GH, MPA had an indirect influence on math (.003, *p* = .000), reading (.001, *p* = .000), and science (.002, *p* = .000). Meanwhile, SWB mediated the effects of MPA on math (.001, *p* = .000), reading (.002, *p* = .000), and science (.001, *p* = .000). To put this number in broader context, OECD (2016) reported .25 to .30 standard deviations as the amount of expected learning gains per year among middle-to-high income countries, while Evans and Yuan (2019) estimated learning gains per year between .15 and .21 standard deviations among low-to-middle income countries. Based on these prior estimates, conservative calculations indicated that our findings of .036 standard deviations increase in student learning outcomes approximately corresponded to between 1.4 and 1.8 additional months of learning in a typical 9-month school year.

The fulfillment of the hypothesis and contributions of this study are discussed as follows. First, since PISA 2018 dataset included information on students’ MPA and cognitive test results for math, reading, and science, this study managed to utilize SEM to evaluate the relationship between MPA and CF. Our analysis indicated that adolescents’ MPA had positive and significant direct effects on CF, which was consistent with previous research highlighting the role of physical activities in improving academic performance (Bedard et al., 2019; Barbosa et al., 2020).

Second, this study conceptualized students’ CF as their math, reading, and science achievements in PISA 2018. Through our analysis, it turned out that the effects of MPA were positive and significant on all the three subjects. In contrast, previous studies have reported inconsistent findings. For instance, Spruit et al. (2016) found positive effects of physical activities on science, including math, but not on language. While Álvarez-Bueno et al. (2016) reported positive effects on both language and math. Using a large and cross-cultural sample, this study reexamine inconclusive findings in existing literature.

This study also examined two potential mediation factors, adolescents’ GH and SWB, were found to mediate the effects of MPA on CF. By utilizing SEM, this study evaluated the extent to which SWB and GH mediated their relationship. These results have deepened to the understandings on the indirect effects of MPA on adolescents’ academic performance discussed in previous research (Sneck et al., 2019).

This study has the following implications: on the methodological level, it advanced the current discussions on MPA and CF. It used large and cross-cultural data, to study the relationship between MPA and adolescents’ CF and how GH and SWB mediate it. On the policy level, it reminds policymakers that it is beneficial to encourage MPA in schools; also, in order to improve adolescents’ CF, and school performance, educational stakeholders, including governments, schools, community, and parents, should work together to improve adolescents’ GH and SWB are important mechanism factors to consider.

This study mainly depended on the large, statistical data. Future research can go deep into the mechanism to explore the physiological foundations of the model. Pedagogical research is also needed to study how curriculum and educational reforms may benefit from these findings. The reasons behind differences among the subjects were not fully explored in the current study, which also requires further investigation.

## Data Availability Statement

Publicly available datasets were analyzed in this study. This data can be found online at: https://www.oecd.org/pisa/data/2018database/; OECD:PISA 2018 Database.

## Author Contributions

XLuan organized the team, managed the research project, and participated in the analysis of data and the preparation of manuscript. JL conceived and designed the study. XLuo did the literature review, participated in the analysis of data, and managed the corresponding issues. All authors contributed to the article and approved the submitted version.

## Funding

This work was supported by Researchers’ Start-up Fund of Shaanxi Normal University (funding No. 1301032077).

## Conflict of Interest

The authors declare that the research was conducted in the absence of any commercial or financial relationships that could be construed as a potential conflict of interest.

## Publisher’s Note

All claims expressed in this article are solely those of the authors and do not necessarily represent those of their affiliated organizations, or those of the publisher, the editors and the reviewers. Any product that may be evaluated in this article, or claim that may be made by its manufacturer, is not guaranteed or endorsed by the publisher.

## Footnotes

^1^ https://www.oecd.org/pisa/data/2018database/
